# Following up users of a pharmacy-based screening and brief intervention service

**DOI:** 10.1186/1940-0640-8-S1-A41

**Published:** 2013-09-04

**Authors:** Janet Krska, Elizabeth Stokes, Adam Mackridge

**Affiliations:** 1 Medway School of Pharmacy, Universities of Greenwich and Kent, Chatham Maritime, Kent, UK; 2 School of Pharmacy and Biomolecular Sciences, Liverpool John Moores University, Liverpool, UK

## 

Pharmacy-based alcohol screening and brief intervention (SBI) services in England vary in follow-up requirements and achieving follow-up can be challenging. Little is known about service-user views and this study aimed to explore these as well as the feasibility of follow-up. The study was funded by Liverpool Primary Care Trust using an unrestricted educational grant from Lundbeck Limited. Five pharmacies providing alcohol SBI services were purposively selected across three areas. Forty individuals receiving SBI interventions were invited by pharmacy staff to participate and contact details recorded with consent. A researcher conducted semi-structured telephone interviews at 1-2 weeks and 3 months after service use. Interview 1 covered views on the service and interview 2 covered behaviour changes, including subsequent discussions with third parties. Institutional ethics approval was obtained.

Sixteen service users took part in the first follow-up (40%); all viewed it positively and ‘a good idea’, although one felt services should be targeted to under-50s. Not all regarded it as personally relevant and one admitted dishonesty in his responses. However, all felt at ease having a discussion in the consultation room about drinking. Fourteen of the 16 (86%) agreed to three-month follow-up. Of these, six reported talking to others about their own drinking habits and three had discussed the service with family and friends, of whom two had recommended they should make use of it. Two had made significant changes to lifestyle that they attributed to the pharmacy intervention. Pharmacy alcohol services were well received by service users interviewed, a significant proportion of whom had subsequently discussed the pharmacy interaction and/or alcohol use with other people, potentially leading to a cascade effect. Two of 16 interviewees reported significant lifestyle changes at 3 months, which tallies with findings from services based elsewhere. Good follow-up rates were achieved at 2 weeks and three months.

